# The Detrimental and Beneficial Functions of Macrophages After Cochlear Injury

**DOI:** 10.3389/fcell.2021.631904

**Published:** 2021-08-11

**Authors:** Yuan Zhang, Yiyuan Li, Xiaolong Fu, Pengjun Wang, Qin Wang, Wei Meng, Tian Wang, Jianming Yang, Renjie Chai

**Affiliations:** ^1^MOE Key Laboratory for Developmental Genes and Human Disease, Jiangsu Province High-Tech Key Laboratory for Bio-Medical Research, School of Life Sciences and Technology, Southeast University, Nanjing, China; ^2^Department of Otolaryngology Head and Neck, Nanjing Tongren Hospital, School of Medicine, Southeast University, Nanjing, China; ^3^Co-innovation Center of Neuroregeneration, Nantong University, Nantong, China; ^4^Department of Otorhinolaryngology, Affiliated Sixth People’s Hospital of Shanghai Jiao Tong University, Shanghai, China; ^5^Department of Otolaryngology-Head and Neck Surgery, The Second Xiangya Hospital, Central South University, Changsha, China; ^6^Department of Otorhinolaryngology, The Second Affiliated Hospital of Anhui Medical University, Hefei, China; ^7^Institute for Stem Cell and Regeneration, Chinese Academy of Sciences, Beijing, China

**Keywords:** macrophages, cochlear injury, auditory structure, sterile inflammation, immune responses

## Abstract

Macrophages are the main intrinsic immune cells in the cochlea; they can be activated and play a complicated role after cochlear injury. Many studies have shown that the number of macrophages and their morphological characteristics within the major cochlear partitions undergo significant changes under various pathological conditions including acoustic trauma, ototoxic drug treatment, age-related cochlear degeneration, selective hair cell (HC) and spiral ganglion neuron (SGN) elimination, and surgery. However, the exact role of these macrophages after cochlear injury is still unclear. Regulating the migration and activity of macrophages may be a therapeutic approach to reduce the risk or magnitude of trauma-induced hearing loss, and this review highlights the role of macrophages on the peripheral auditory structures of the cochlea and elucidate the mechanisms of macrophage injury and the strategies to reduce the injury by regulating macrophage.

## Introduction

Macrophages are cellular components of the innate immune system and have the hallmarks of heterogeneity and plasticity, and they play important roles in homeostasis, repair, and pathological changes. They are mainly transformed by monocytes from circulation and reside in virtually all tissues. In general, there are three main sources of macrophages in adult tissues in the absence of external stimuli, namely, fetal liver monocytes, yolk sac macrophages, and bone marrow (BM)-derived monocytes (Ginhoux and Guilliams, 2016). Macrophages can sense the microenvironment and differentiate into two distinct phenotypes, which consist of M1 and M2 subsets. M1 are called classically activated or inflammatory macrophages and M2 are called alternatively activated or wound-healing macrophages (Mills and Ley, 2014).

**FIGURE 1:**
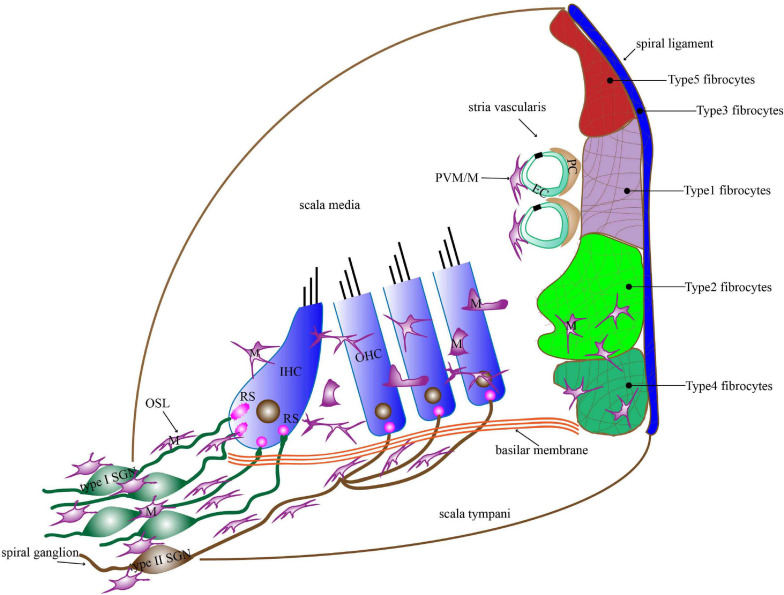
The distribution of macrophages in the basilar membrane, spiral ligament, stria vascularis, OSL, and spiral ganglion. Macrophages in the basilar membrane are distributed below in the HCs on the scala tympani side. Macrophages in the apical turn show dendritic morphology, those in the middle turn display irregular morphology with short projections, and those in the basal turn transform into amoeboid morphology. Macrophages in the lateral wall contain PVM/Ms of the stria vascularis and macrophages among fibrocytes in the spiral ligament. Macrophages are present around the neural tissue, which is composed of SGN cell bodies and their peripheral nerve fibers inside the OSL as well as the modiolus. Most of the macrophages in the OSL area lie in a direction parallel to the radial fibers. M, macrophages; PVM/M, perivascular macrophage-like melanocyte; RS, ribbon synapse; EC, endothelial cells; PC, pericytes; IHC, inner hair cell; OHC, outer hair cell; OSL, osseous spiral lamina; SGN, spiral ganglion neuron.

Macrophages are the main executive cells of the immune system in the cochlea. They reside in multiple anatomic sites including the basilar membrane, osseous spiral lamina (OSL), spiral ganglion, spiral ligament, and stria vascularis (Hirose et al., 2005; Okano et al., 2008; Sato et al., 2008; Shi, 2010; Figure 1). Although the exact function of these macrophages is unclear, their widespread distribution in the cochlea implies that they participate in maintaining cochlear homeostasis and preventing diseases. Indeed, many studies have shown that immune system contribute to the development of noise-related hearing loss, drug-related hearing loss, and age-related hearing loss (Yang et al., 2015; Frye et al., 2017; Kaur et al., 2018). Cochlear implant surgery, currently the only effective method for treating severe sensorineural hearing loss (SNHL), can also trigger an immune response (Bas et al., 2015). As the main specialized immune cells in the cochlea, the number of macrophages and their morphological characteristics at various cochlear locations undergo significant changes, and the local environmental factors that facilitate macrophage differentiation and behavior are also significantly altered after cochlear damage, which supports the idea that macrophages play a key role in response to cochlear injury (Hu et al., 2018).

Macrophages migrate into the cochlea in response to damage caused by noise exposure, ototoxicity, surgery, or age-related degeneration. Damage-associated molecular patterns (DAMPs) produced by injured tissue bind to pattern recognition receptors (PRRs) expressed on the macrophages, and this activates resident macrophages to secrete pro-inflammatory cytokines, leading to apoptosis of injured cells and promoting further infiltration of immune cells (Frye et al., 2019). However, some studies suggest that the recruitment of macrophages can promote the survival of spiral ganglion neurons (SGNs) and the spontaneous recovery of ribbon synapses after cochlear injury (Kaur et al., 2015, 2018, 2019). Moreover, some studies demonstrate that macrophages can engulf dead cells and cellular debris in the organ of Corti and promote the regeneration of hair cells (HCs) in damaged sensory epithelium (Carrillo et al., 2016). These results suggest that macrophages not only play a damaging role, but also play a protective role after cochlear injury (Table 1).

**TABLE 1:** The role of macrophages on the hair cell (HC), spiral ligament, stria vascularis, ribbon synapse, and spiral ganglion neuron (SGN) in different injury models.

	**Macrophage function**				
**Injury Model**	**HC**	**Spiral ligament**	**Stria vascularis**	**Ribbon synapse**	**SGN**
Noise exposure	HC degeneration and phagocytosis	Pro-inflammatory role	Maintain integrity of BLB	Promote recovery	Promote survival
Ototoxic drug treatment	HC degeneration, phagocytosis, and regeneration	Pro-inflammatory role			Promote survival
Aging	HC degeneration and phagocytosis		Maintain integrity of BLB		
Selective HC elimination	HC phagocytosis				Promote survival
Selective SGN elimination					Repair
Cochlear implantation	Anti-inflammatory role	Pro-inflammatory role			Anti-inflammatory

To understand the precise role of cochlear macrophages after aseptic injury, we firstly review the origin, the distribution, and the influence of macrophages on the peripheral auditory structures of the cochlea. We then describe the changes of cochlear macrophages after aseptic injury, the signaling pathways leading to the activation of macrophages, and the secretion of inflammatory cytokines in response to damage. Finally, we present the currently available methods for inhibiting macrophage-induced damage.

## The Origin of Cochlear Macrophages

Although the inner ear was once considered as an immune privileged organ, recent studies have shown that macrophages are present in the inner ear and play critical roles in the immune response of the inner ear. Resident macrophages of the cochlea may have different origins according to the stages of development. In the early development of the mouse cochlea, macrophages arise *via* hematopoiesis in the yolk sac. Macrophages first appear at about embryonic day (E) 10.5 in the developing otocyst and then gradually migrate into the mesenchyme of the cochlea during the embryonic stage and subsequently take up residence especially in the postnatal cochlear spiral ligament, the stria vascularis, and the spiral ganglion. The density of mouse cochlear macrophages increases with growth, reaches a peak around the neonatal stages, and decreases from postnatal day 3 (P3). In addition, macrophages in the cochlea have the ability to proliferate *in situ*, especially during the perinatal period (Kishimoto et al., 2019). In the adult mouse cochlea, tissue-resident macrophages are gradually replaced by BM-derived cells over the course of several months in the steady state (Okano et al., 2008; Sato et al., 2008; Shi, 2010). Therefore, the number of macrophages can change rapidly after cochlear injury to protect against or aggravate cochlear tissue injury.

## Distribution of Tissue Macrophages in Mature Cochlear Tissues

### Macrophages in the Basilar Membrane

The basilar membrane contains two sets of macrophages—one on the organ of Corti side and the other on the scala tympani side—and these two sets show different developmental patterns and fates after birth. At birth, macrophages on the organ of Corti side are morphologically completely differentiated. However, they have a very short life span and undergo developmental deterioration as the sensory epithelium matures (Dong et al., 2018). Thus, immune cells in the organ of Corti are absent under homeostatic conditions (Hirose et al., 2005; Okano et al., 2008; Du et al., 2011; Yang et al., 2015). Macrophages on the scala tympani side are round shape at P1 and begin site-specific differentiation at P4. Macrophages in the apical turn show dendritic morphology, while macrophages of the middle and basal turns have irregular morphologies with short projections, and macrophages of the basal turn can further differentiate and transform into an amoeboid morphology (Dong et al., 2018).

The entire length of the basilar membrane contains macrophages, and this is the case in a variety of species such as mice, chickens, zebrafish, and humans (Hirose et al., 2005; Warchol et al., 2012; O’Malley et al., 2016). These macrophages are the closest immune cells to sensory cells in terms of physical distance and can sense pathological changes in sensory cells. There are two different extracellular environments surrounding these macrophage populations, with one surface contacting the basement membrane and mesothelial cells and the other surface contacting the perilymph, and these different environments allow the cells to monitor the immune environments within the organ of Corti and perilymph. Moreover, macrophages are less restricted physically due to the acellular environment of scala tympani, and thus they change their morphology and migrate more easily (Hu et al., 2018).

### Macrophages in the Lateral Wall

There are two types of macrophages in the lateral wall of the cochlea, namely, macrophages in the spiral ligament and perivascular macrophage-like melanocytes (PVM/Ms) in the stria vascularis (Zhang et al., 2012). Macrophages in the spiral ligament are irregularly shaped with branches and processes and they are distributed abundantly on the inferior site of spiral ligament (Wang et al., 2002; Hirose and Liberman, 2003). The inferior site is adjacent to the scala tympani, which is where leukocytes mainly accumulate after acute injury (Sautter et al., 2006; Du et al., 2011). Morphological analysis has also demonstrated that the surface of the inferior site contains micropores (Lim, 1970), and these microcellular structures allow the perilymph to enter the spiral ligament, thereby enabling communication between the perilymph and the macrophages of the spiral ligament.

The cochlea has a blood–labyrinth barrier (BLB), which maintains the microenvironment of the cochlea and separates the blood vessels of the stria vascularis from systemic circulation. The BLB is constituted by endothelial cells, a large number of PVM/Ms with both macrophage and melanocyte characteristics, pericytes, and the underlying basement membrane (Zhang et al., 2012). The PVM/Ms are in close contact with blood vessels through cytoplasmic processes, and they wrap around endothelial cells and pericytes to form structures that are essential for maintaining normal capillary architecture (Shi, 2010).

### Macrophages in the Neural Tissues

Macrophages are present around the neural tissue of the cochlea, which is composed of SGN and their peripheral nerve fibers inside both the OSL and the modiolus. Macrophages distributed in the OSL region are roughly arranged into two rows—one near the SGNs and the other near the edge of the OSL. Although most of these macrophages lie in a direction parallel to the radial fibers of the tunnel, some macrophages are vertically oriented. These cells can extend their processes to the region of inner HCs through the habenula perforata without contact with inner HCs. Although the function of these macrophage processes is not fully understood, their adjacency to cochlear ribbon synapses suggests a role in removing degraded sensory cells in the organ of Corti after injury and maintaining synapse homeostasis, a function that has been identified in microglia, which are the resident macrophages in the central nervous system (CNS) (Wake et al., 2009; Hirose et al., 2017).

### Chemokine Signaling Recruits Macrophages Into Cochlear Tissue

Cochlear injury results in the rapid accumulation of macrophages, but identifying the signaling pathways mediating this recruitment has proven to be challenging. The main reason for this is that cochlear damage includes not only HCs, but also other types of cells in the cochlea such as supporting cells, lateral wall fibrocytes, and SGNs, and these cells might generate their own signaling molecules for recruiting macrophages. So far, two chemokine signaling pathways that recruit macrophages have been studied in the cochlea.

Mononuclear phagocytes consist of monocytes, dendritic cells, microglia, and tissue macrophages, and these cells can be divided into two distinct populations (Geissmann et al., 2003). One population is the CX3CR1-expressing mononuclear phagocytes, the majority of which are tissue macrophages such as those in the cochlea and microglia in the CNS (Jung et al., 2000). The only ligand for CX3CR1 is CX3CL1, also known as fractalkine. Neurons and endothelial cells, including SGNs and HCs, express high levels of CX3CL1 (Sun et al., 2015), which can stimulate and signal macrophages through the CX3CR1 receptor after cochlear injury, thus regulating the adhesion and migration of macrophages (Limatola and Ransohoff, 2014). The other population is the CCR2-expressing monocytes. CCR2 binds to its ligand CCL2 and plays a unique role in recruiting mononuclear cells (Lu et al., 1998). Although CCR2-expressing monocytes do not remain as resident cells in peripheral organs for long periods, they can transdifferentiate and act as a precursor for resident macrophage (Bain et al., 2014). In addition, CCR2 plays a crucial role in the release of monocytes into the circulation from the BM (Serbina and Pamer, 2006). Once in circulation, CCR2-expressing monocytes can migrate from the blood circulation to peripheral tissues, even though cells of the inner ear do not express CCL2 or CCR2 (Sautter et al., 2006; Hirose et al., 2014).

Important distinctions have been found between CX3CR1 and CCR2 macrophages not only in terms of their surface markers, but also in terms of their effector function in immune responses (Hirose and Li, 2019). CX3CR1-expressing cells become numerous in the inner ear after excessive noise exposure and in response to aminoglycoside ototoxicity, while CCR2-expressing cells enter the cochlea after lipopolysaccharide exposure and other forms of cochlear damage, such as bacterial meningitis (Hirose et al., 2014).

## The Role of Macrophages in Different Structures of the Cochlea

### Macrophages Play a Complicated Role in the Basilar Membrane

#### Sensory Cell Degeneration

Macrophages are immune sensors that detect changes in the microenvironment of the organ of Corti, and mature tissue macrophages can influence the pathogenesis of the sensory cell and the degeneration of sensory cells in the early stage (Yang et al., 2015; Frye et al., 2017; Zhang et al., 2017). Sensory cells are completely degraded by 10 weeks after amikacin treatment, while the macrophage density increases significantly over the same time period (Ladrech et al., 2007). Macrophages trigger inflammation by releasing inflammatory cytokines in response to cochlear sensory epithelium injury (Sun et al., 2015), and cochleae with greater numbers of outer HC lesions have significantly more mature tissue macrophages than cochleae with less outer HC lesions (Yang et al., 2015; Frye et al., 2017). Together, these observations suggest that macrophages are involved in epithelial degeneration.

### Phagocytosis of HC Debris

Macrophages play a role in removing waste products from the organ of Corti (Wang and Li, 2000). Because there are no resident macrophages in the organ of Corti under physiological conditions, macrophages outside of the organ of Corti can stretch out their processes into the region of inner HCs in order to eliminate cellular debris (Hirose et al., 2017). In addition, macrophages can migrate into the organ of Corti in severely injured cochlea in which the overall structure of the organ of Corti is destroyed (Fredelius and Rask-Andersen, 1990), and macrophages have been observed engulfing degenerated cells and debris in the tunnel of Corti and outer HC region 5 days after acoustic injury (Fredelius and Rask-Andersen, 1990).

### HC Regeneration

Several studies have demonstrated that macrophages release signals that influence the early stages of HC regeneration in the damaged sensory epithelium. It has been shown that an increase in the number of macrophages precedes the proliferation of HC precursors in the damaged sensory epithelium of the avian inner ear after treatment with aminoglycosides (Bhave et al., 1998). In addition, the latency with which macrophages are recruited into injured regions is about 4–8 h, which implies a role of macrophages in the initiation of HC regeneration (Warchol, 1997). Finally, the regeneration of HCs is significantly delayed by the genetic ablation of macrophages that would normally be recruited to the region of injury or local ablation of these macrophages using clodronate liposomes (Carrillo et al., 2016).

### Two Types of Macrophages Play Different Roles in the Lateral Wall

#### Macrophages of the Spiral Ligament Are Responsible for Inflammatory Responses

Bone marrow-derived resident macrophages exist in the spiral ligament and can be activated by various kinds of damage, such as noise exposure, ototoxic drug application, and surgical stress. These macrophages communicate with spiral ligament fibrocytes and are responsible for the inflammatory response (Hirose et al., 2005), which is detrimental to hearing recovery (Fujioka et al., 2014).

#### PVM/Ms in the Stria Vascularis Maintain the Integrity of the BLB

Macrophages play an role not only in immunity but also in mediating the integrity and permeability of the BLB in the stria vascularis (Zhang et al., 2012; Neng et al., 2013). Infiltrating macrophages migrate to BLB to update PVM/Ms, and noise exposure can accelerate the circulation of PVM/Ms (Dai et al., 2010; Shi, 2010). The absence of PVM/Ms results in an increase in BLB permeability to both low and high molecular weight tracers (Zhang et al., 2012). PVM/Ms are also critical for establishing and maintaining the endocochlear potential (Zhang et al., 2013), and PVM/M-depleted animals show a substantial drop in endocochlear potential accompanied by hearing loss (Zhang et al., 2012). In addition, stria vascularis degeneration defined as hyperpigmentation is related to macrophage invasion (Jabba et al., 2006). Taken together, these observations suggest that PVM/Ms alter the BLB not only through immune responses, but also *via* physical and molecular dysfunctions that are different from macrophages in other regions of the cochlea.

### Macrophages Promote Ribbon Synapse Development and Recovery After Injury

Recent studies have suggested that macrophages may promote the development of ribbon synapses in the cochlea. Ribbon synapses are the locations where glutamate is released from HCs onto the peripheral afferent terminals of SGNs in response to sound input. In the CNS, microglia cells take part in the formation and remodeling of synapses, and they can remove weakly active synapses and do so in response to factors such as CX3CL1 (Schafer and Stevens, 2015; Wu et al., 2015). Some studies have shown that macrophages in the cochlear OSL might have similar effects to the microglia in the CNS. Macrophages in the OSL are present in early development after birth and near the ribbon synapses (Dong et al., 2018), which suggests a role for these cells in ribbon synapse formation. Meanwhile, the number of macrophages is highest between P1 and P10 (Dong et al., 2018), corresponding to the initial period of ribbon synapse formation and pruning (Nemzou et al., 2006; Huang et al., 2012). Importantly, macrophages appear to enhance functional ribbon synapse recovery after noise injury (Kaur et al., 2019).

### Macrophages Protect Auditory Nerve After Injury

Recent studies have identified the important role of macrophages in SGN protection (Kaur et al., 2015, 2018, 2019). BM-derived macrophages are involved in the repair of the auditory nerve after injury to SGN and are important resources for promoting auditory nerve remodeling in the adult cochlea (Lang et al., 2016). Cochlear macrophages remove redundant glial cells during the development of the auditory nerve, and depletion of macrophages leads to an increase in the number of glial cells, abnormal formation of myelin sheaths, and impaired hearing functions (Brown et al., 2017). Macrophages also help to clear SGNs undergoing apoptosis during early postnatal stages, which is the time of ribbon synapse development (Echteler et al., 2005). Moreover, macrophages have been demonstrated to prevent loss of SGNs when the cochlea is injured (Kaur et al., 2015, 2018).

## Changes and Effect of Macrophages on Cochlear Structure Caused by Cochlear Injury

### Macrophages in Noise-Induced Hearing Loss

Acoustic trauma can activate the immune defense function of the cochlea (Yang et al., 2016). At present, two noise exposure levels have been used to investigate macrophages in the cochlea. Lower level noise (LLN) is that which causes only temporary threshold shifts without HC loss, while higher level noise (HLN) is that which causes permanent threshold shifts accompanied by HC loss. Both LLN and HLN can rapidly increase the number of macrophages and lead to alterations in macrophage shape in the basilar membrane, OSL, and spiral ganglion (Frye et al., 2018; Kaur et al., 2018). Studies have shown that the phagocytic ability of macrophages is reduced and that the activated macrophage morphology does not fully recover until 2 months after LLN exposure (Frye et al., 2018). In addition, the expression of the major histocompatibility complex class II (MHC II) antigen-presenting protein by basal monocytes and macrophages increases after HLN. CD4^+^ T cells also infiltrate into the same region after noise exposure, suggesting that the activation of the antigen presentation function is site-dependent, and these cells connect innate immunity to the adaptive immune response (Yang et al., 2015).

CX3CR1 and CCR2 knockout have no significant effect on the number of macrophages in the sensory epithelium or spiral ganglion after acoustic injury (Sautter et al., 2006; Sato et al., 2008; Kaur et al., 2018), nor does the absence of CX3CR1 in mice have any striking effect on HCs or spiral ligament injuries or on hearing thresholds after HLN exposure (Sato et al., 2008). However, noise-induced HC death is significantly increased in the CCR2 knockout mice. This observation suggests that CCR2 may have a protective effect in the cochlea after acoustic injury (Sautter et al., 2006).

Noise exposure causes activation of PVM/Ms and destroys the BLB (Shi, 2009; Yang et al., 2011; Zhang et al., 2013). Bone marrow-derived cells (BMDCs) migrate to the BLB areas of the damaged cochlea in the first week after noise exposure and further accumulate in the second week. After 4 weeks, the BMDCs become fused with blood vessels (Dai et al., 2010). Activated PVM/Ms show reduced contact with capillaries and a great distance from pericytes and endothelial cells (Zhang et al., 2013; Jiang et al., 2019), and they produce less pigment epithelium growth factor, which is crucial for stabilizing the BLB and for maintaining normal hearing functions (Liu et al., 2012; Zhang et al., 2012).

**FIGURE 2:**
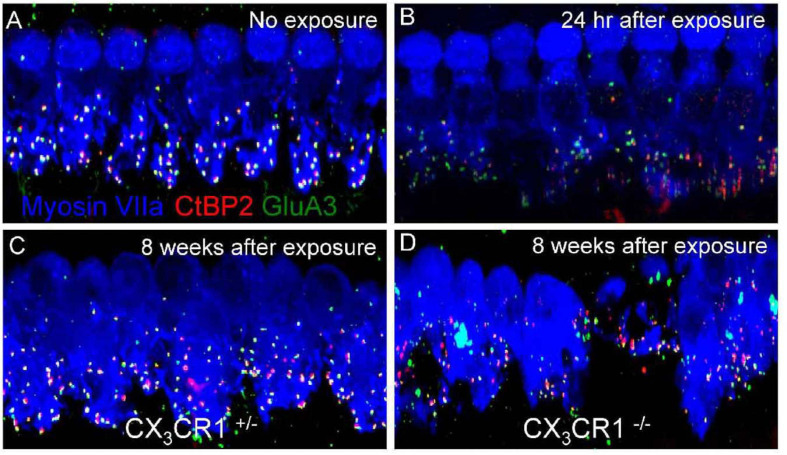
Ribbon synapses in CX3CR1C^+/–^ and CX3CR1^– /–^ mice after noise exposure. Representative micrographs are shown for the 32-kHz region. **(A)** CX3CR1C^+/–^ mice without noise exposure show intact ribbon synapses. **(B)** CX3CR1C^+/–^ mice at 24 h after exposure show disintegrated ribbon synapses. **(C)** CX3CR1C^+/–^ mice at 8 weeks after exposure show repaired synapses. **(D)** CX3CR1^– /–^ mice at 8 weeks after exposure show degenerated ribbon synapses. The pre-synaptic marker CtBP2 is in red, the post-synaptic marker GluA3 is in green, and myosin VIIa is in blue. Scale = 17 mm [citing from Kaur et al. (2019)].

Although the mechanism is still unclear, macrophages and CX3CR1 signaling are suggested to contribute to ribbon synapse recovery in response to damage. LLN leads to rapid ribbon synapse degeneration, with macrophages immediately migrating into the injured synaptic region. The injured synapses can spontaneously recover in animals with intact CX3CR1 signaling. However, a lack of CX3CR1 signaling reduces synaptic recovery and increases neuronal loss after noise exposure (Kaur et al., 2019; Figure 2).

### Macrophages in Ototoxic Drug-Induced Hearing Loss

Recent studies have focused mainly on the effect of ototoxic drugs on the number of macrophages and on how CX3CR1 expressed on macrophages regulates the effects of ototoxic drugs on cochlear injury. Kanamycin/furosemide treatment increases the numbers of macrophages in the sensory epithelium, spiral ganglion, and spiral ligament (Hirose et al., 2014; Kaur et al., 2018). CX3CR1 deficiency results in a decrease in macrophages in the sensory epithelium and has no impact on the recruitment of macrophage into the spiral ganglion, but leads to increased SGN loss (Kaur et al., 2018). This implies that CX3CR1 may have distinct roles on the cochlear epithelium or the spiral ganglion after kanamycin/furosemide exposure.

Neomycin increases the expression of CX3CL1 on HCs and the expression of CX3CR1 on macrophages in the basilar membrane, and CX3CL1 activates macrophages and increases cytokine levels in the cochlea. A lack of CX3CR1 results in the survival of significantly greater numbers of cochlear HCs after neomycin treatment *in vitro*, which suggests that CX3CL1/CX3CR1-mediated macrophage activation has a damaging effect on HCs (Sun et al., 2015). However, another study showed that the absence of CX3CR1 on macrophages leads to more severe hearing loss and exacerbates HC loss after kanamycin exposure. In addition, macrophages are more concentrated in the high-frequency region of the cochlea and are more abundant in the spiral ligament after CX3CR1 knockout (Sato et al., 2010). These discrepancies suggest that further exploration of the effect of CX3CR1 on HCs in the cochlea is needed.

### Macrophages in Age-Related Hearing Loss

The development of presbycusis is affected by immune function (Iwai et al., 2003), and age-related immune system dysfunction can accelerate presbycusis (Iwai et al., 2008). At present, the research in this area is mainly focused on changes in basilar membrane macrophages and PVM/Ms in the aging cochlea.

There is no mass inflammatory cell infiltration in the basilar membrane of the aging cochlea, and in fact, the macrophage numbers decrease with age (Frye et al., 2017). The main immune cell reaction is inflammatory activation of resident mature macrophages characterized by morphological changes, including increased size and transformation into an amoeboid form (Frye et al., 2017; Zhang et al., 2017). Macrophage activation precedes the onset of sensory cell pathogenesis, and there is a morphological transition from branching to more round or amoeboid morphology in the areas that are undergoing active sensory cells pathogenesis. However, when the degeneration of sensory cells is completed, the number of macrophages decreases and the morphology of the macrophages returns to a resting state in that area.

In the stria vascularis of the aging cochlea, PVM/Ms show a significant decrease in density and size, but a significantly greater amount of melanin. Most of the PVM/Ms are branched and are arranged in a self-avoidance pattern in the younger cochlea, while the PVM/Ms are flattened and amoeboid and show less physical contact with the capillaries in some regions of the older cochlea (Neng et al., 2015).

### Macrophages in the Cochlea After Selective HC or SGN Elimination

**FIGURE 3:**
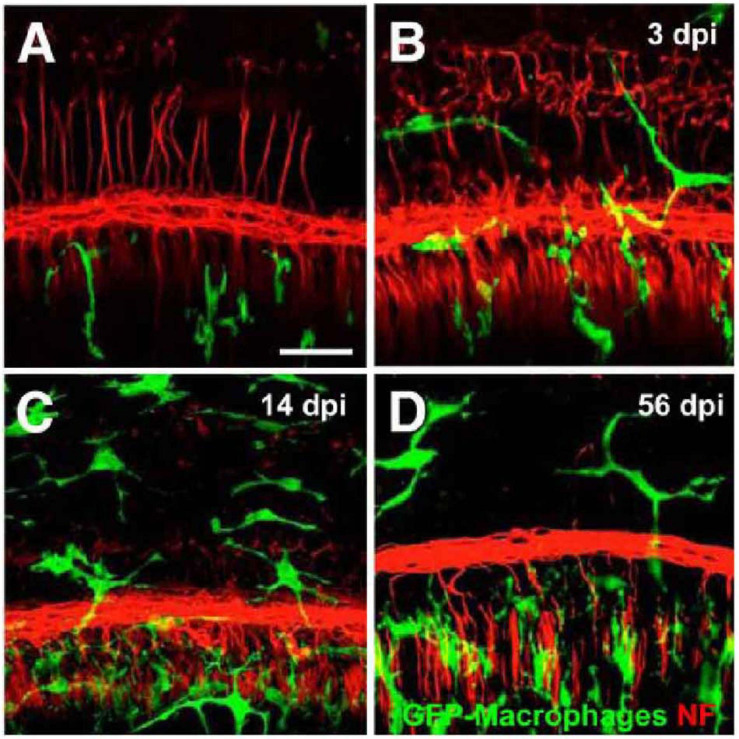
Macrophages in the sensory epithelium of the cochlea after HC loss. Pou4f3^+/+^ (control) and Pou4f3^*DTR/*+^ mice were injected with a single dose of DT, and cochlear whole mounts were examined. **(A)** Controls, **(B)** 3 days after DT, **(C)** 14 days after DT, **(D)** 56 days after DT. GFP-expressing macrophages are in green, and neurons (neurofilaments) are in red. Compared with controls, the numbers of macrophages increased in the sensory epithelium of damaged (Pou4f3^*DTR/*+^) mice. Scale bar = 30 μm [citing from Kaur et al. (2015)].

**FIGURE 4:**
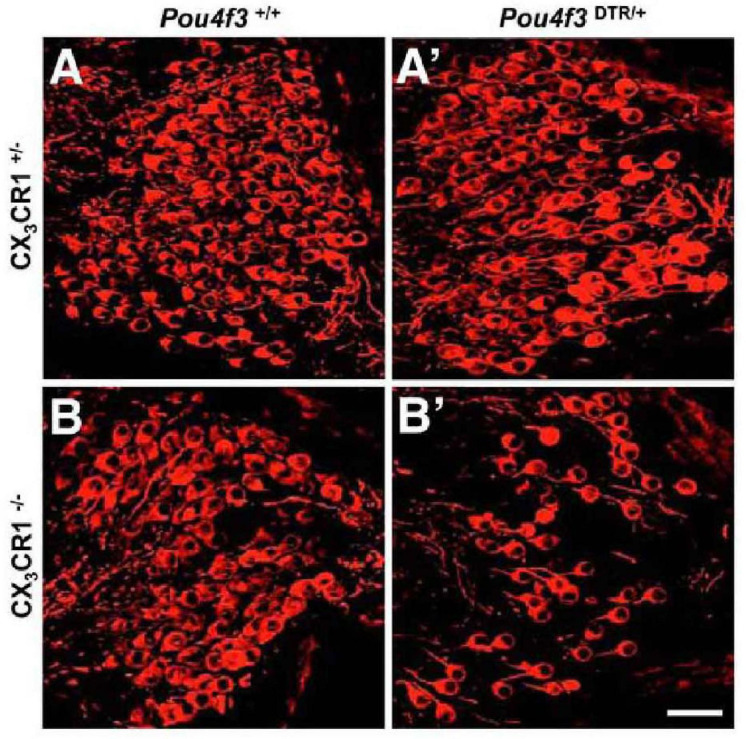
CX3CR1 deficiency leads to significant SGN loss after HC ablation. **(A)** CX3CR1C^+/–^ mice without DT, **(B)** CX3CR1^– /–^ mice without DT, **(A′)** CX3CR1C^+/–^ mice at 56 days after DT, **(B′)** CX3CR1^– /–^ mice at 56 days after DT. Compared with CX3CR1C^+/–^ mice, significant SGN loss in cochlear mid-modiolar sections of CX3CR1^– /–^ mice was observed. No SGN loss was seen in Pou4f3^+/+^ mice that were either CX3CR1^– /–^ or CX3CR1C^+/–^. Neurons (neurofilaments) are in red. Scale bar = 30 μm [citing from Kaur et al. (2015)].

Damage to HCs or SGNs alone is sufficient to recruit macrophages into the cochlea. Pou4f3 is a HC-specific transcription factor, and the application of diphtheria toxin (DT) results in almost complete removal of cochlear HCs in mice selectively expressing human DT receptor under Pou4f3 control, with no significant pathological changes in the supporting cells, SGNs, or cochlear lateral wall cells (Kaur et al., 2015). The death of HCs leads to an increase in basilar membrane and spiral ganglion macrophages (Kaur et al., 2015; Figure 3). The number of basilar membrane macrophages peaks at 14 days after HC loss and then begins to decrease, while the number of spiral ganglion macrophages remains elevated for at least 56 days. When CX3CR1 signaling is disrupted, the number of recruited macrophages in the sensory epithelium and spiral ganglion is reduced and cochlear SGN loss increases (Kaur et al., 2015; Figure 4).

Ouabain inhibits Na/K-ATPase activity, and applying ouabain to gerbils and mice *via* the round window can rapidly and highly selectively eliminate type I SGNs without leading to cellular degeneration in the organ of Corti or the cochlear lateral wall (Schmiedt et al., 2002; Lang et al., 2005, 2010). The number of macrophages in the spiral ganglion increases 3 days after ouabain exposure (Lang et al., 2016). The expression of genes that are linked to macrophage activation is upregulated, and the infiltrating macrophages show an amoeboid or large round shape, indicating that the macrophages have reactive or engulfment functions.

### Macrophages in the Cochlea Following Cochlear Implantation

The phagocytic and anti-inflammatory activities of macrophages are both enhanced after cochlear implantation (Bas et al., 2015; Okayasu et al., 2020). In a study of patients with unilateral cochlear implantation, activated and phagocytosing macrophages were found in both the fibrotic sheath around the electrode track and the fibrous tissue with infiltrating lymphocytes in the implanted ears, and macrophage densities in the OSL and Rosenthal’s canal in the implanted ears were markedly higher than in the unimplanted ears (Okayasu et al., 2020). The insertion of an electrode array into the mouse cochlea induces increased macrophage infiltration with M1 macrophages playing a main role in the spiral ligament and M2 macrophages playing a main role in the organ of Corti and spiral ganglion, and both M1 and M2 macrophages are present at the wound site around the inserted electrode (Bas et al., 2015).

### Macrophage Activation Signaling in Response to Tissue Injury

Macrophages have a protective effect on synapses and SGNs, but the mechanisms are still unknown. Numerous studies have shown that activated macrophages produce inflammatory mediators that are involved in cochlear injury. At present, most studies on cochlear macrophages are performed in aseptic injury models, which suggest that any observed cochlear inflammation must be an endogenous response to cellular stress or injury. Sterile inflammation of other organ systems is closely related to the DAMPs that are released by stressed or damaged tissues (Tang et al., 2012; Schaefer, 2014) and that can recognize and activate the PRRs on the surfaces of macrophages. PRR activation can rapidly activate resident macrophages that secrete pro-inflammatory cytokines and produce reactive oxygen species, leading to apoptosis of damaged cells and the infiltration of immune cells (Hume et al., 2001; Park et al., 2004; Tsung et al., 2007).

**FIGURE 5:**
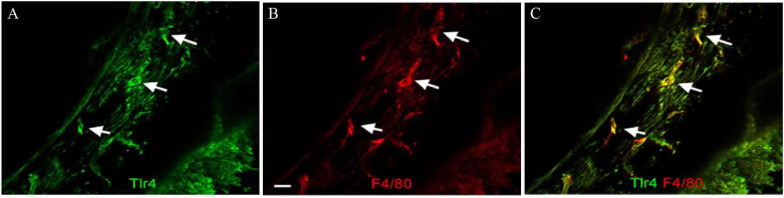
TLR4 expression in macrophages of the basilar membrane. **(A)** TLR4 is expressed in cells of the basilar membrane. Arrows indicate TLR4-positive cells. **(B)** Double labeling of the macrophage marker protein F4/80 in the same tissue. **(C)** Merged images of panels **(A,B)**. The TLR4-positive cells are also F4/80 positive. Scale bar = 20 μm [citing from Vethanayagam et al. (2016)].

Toll-like receptor 4 (TLR4) is one of many PRRs that can be activated in aseptic inflammation, and TLR4 activation can promote the production of reactive oxygen species and the activation of canonical NF-kB (Park et al., 2004; Fan et al., 2007). Several studies have shown that TLR4 activation is one of the pathways that leads to cochlear inflammation after noise exposure or ototoxic drug treatment. Macrophages of the basilar membrane in the cochlea constitutively express TLR4 (Figure 5), but the lack of TLR4 has no impact on hearing functions under normal conditions (Vethanayagam et al., 2016). Although the exact ligand that activates TLR4 after noise exposure or ototoxic drug treatment is unknown, each type of injury leads to significantly increased expression of TLR4 in the cochlea within hours of the injury (Oh et al., 2011; Hirose et al., 2014; Vethanayagam et al., 2016). TLR4 activation caused by noise damage or cisplatin treatment results in NF-kB activation and promotes the production of TNF-α, IL-1β, and IL-6 (So et al., 2007; Oh et al., 2011; Zhang et al., 2019). Cisplatin treatment in TLR4-deficient mice results in reduced inflammation and, in particular, reduced expression of TNF-α expression in mice, which in turn results in reduced hearing loss (Oh et al., 2011). TLR4 knockout mice also show relatively more resistance to noise, and the absence of TLR4 suppresses the expression of MHC-II in macrophages and reduces the antigen presentation activity of macrophages. TLR4 dysfunction inhibits IL-6 production in the organ of Corti (Vethanayagam et al., 2016), while activation of NF-κB is concomitant with the morphological transformation of macrophages, which is further evidence that macrophages are activated *via* TLR4 (Herranen et al., 2018). Together, these studies demonstrate the negative effects that macrophages can have in the inner ear.

## Inflammatory Cytokines Participate in Cochlear Injury

Inflammatory factors are produced at different levels at the different time points, and they are distributed in different anatomical sites. The expression of gene encoding TNF-α is upregulated at 6 h after noise-induced damage, and IL-1β and IL-6 expression increases as early as 3 h after noise damage, while IL-6 expression decreases at 24 h, indicating transient upregulation in the cochlea in response to noise exposure (Fujioka et al., 2006; Wakabayashi et al., 2010; Tan et al., 2016; Mizushima et al., 2017). Cisplatin injection results in an increase in TNF-α, IL-1β, and IL-6, and TNF-α and IL-1β expression is observed throughout the organ of Corti, the spiral ligament, and the stria vascularis in the cochlea. TNF-α inhibitors can not only completely inhibit TNF-α expression but also significantly block IL-1β and IL-6 expression. These observations imply that TNF-α plays a dominant and central role in the pathophysiology of HC damage induced by cisplatin (So et al., 2007).

IL-1β expression is a general cochlear response to trauma (Satoh et al., 2002), and a lack of CX3CR1 can result in enhanced expression of IL-1β in the spiral ganglion after ototoxic or acoustic injury (Kaur et al., 2018). IL-6 and its receptor are present in the stria vascularis, spiral ligament, and spiral ganglion, and IL-6 receptor is also detected in HCs after noise exposure (Fujioka et al., 2006; Wakabayashi et al., 2010). IL-6 is initially expressed in the cytoplasm of Type III and Type IV fibrocytes, and it expands to a broader area throughout the lateral wall, eventually reaching the stria vascularis. Because the macrophage marker Iba-1 staining is not expressed in the IL-6 expression region, the early IL-6 expression might not be due to macrophage activation (Fujioka et al., 2006). HLN exposure induces markedly higher expression of TNF-α, IL-1β, and IL-6 as compared with LLN exposure, which suggest that more severe local inflammation is induced in the HLN group (Shin et al., 2019).

ICAM-1 is an adhesion molecule that mediates leukocyte migration to vascular endothelial cells during inflammation, and ICAM-1 is expressed on fibrocytes in the inferior part of the spiral ligament and in vascular endothelial cells under physiological conditions (Suzuki and Harris, 1995; Tornabene et al., 2006). After HLN exposure, the gene expression of ICAM-1 in the spiral ligament and endosteal cells is upregulated at 6 h, and the protein expression begins at 24 h, subsequently reaching a maximum at around 2–4 days and then returning to basal levels by 14 days (Tornabene et al., 2006; Tan et al., 2016). LLN can also enhance ICAM-1 expression in macrophages of the basilar membrane (Frye et al., 2018).

## Regulation of Macrophage Migration or Function to Improve Hearing

### Inhibition of Macrophage Migration After Cochlear Injury

Inhibition of macrophage migration after cochlear injury prevents hearing loss, HC loss, and damage to SGNs. Macrophages and monocytes can be depleted by clodronate liposomes, and after noise exposure, mice treated with clodronate have decreased macrophage recruitment into the stria vascularis and exhibit significantly reduced hearing threshold shifts and less outer HC loss in the lower-apical cochlear turn (Mizushima et al., 2017). Macrophage migration inhibitory factor (MIF) plays a role in neural development and is detected in the stria vascularis and spiral ligament. Lack of MIF results in significant loss of outer HCs and reduced numbers of SGCs and thus results in profound hearing loss after noise exposure (Kariya et al., 2015). Inducible nitric oxide synthase (iNOS) from PVM/Ms can recruit BMDCs to the cochlear BLB after acoustic injury, and stromal cell-derived factor-1alpha (SDF-1α) and its chemokine receptor 4 (CXCR4) are required for the BMDC recruitment triggered by iNOS. Inhibiting iNOS/SDF-1α signaling reduces the BMDC infiltration required for cochlear vascular repair as evidenced by decreased vascular density (Dai et al., 2010). These findings imply that inhibition of macrophage migration might be a promising therapeutic option for preventing hearing loss after cochlear injury.

### Inhibiting Inflammatory Cytokine Expression by Macrophages or Blocking the Activity of Inflammatory Cytokines

Inhibiting the expression of certain inflammatory cytokines might be an important means of protecting the inner ear after injury. Neomycin can activate macrophages in the basilar membrane *in vivo* or *in vitro* (Sun et al., 2015), and minocycline can inhibit the release of inflammatory cytokines by macrophages (Owolabi and Saab, 2006; Sun et al., 2007; Shan et al., 2011). Inhibiting the activation of macrophages with minocycline can reduce the HC loss caused by neomycin and can improve hearing function in neomycin-treated mice (Sun et al., 2015).

Heat shock transcription factor 1 (HSF1) mainly plays a role in regulating heat shock response, but it also directly or indirectly inhibits inflammatory cytokine expression. IL-1β and IL-6 expression and elevated auditory thresholds after noise exposure are significantly suppressed after administration of geranylacetone, which activates HSF1 (Nakamoto et al., 2012). Acetyl-L-carnitine prevents the increased levels of TNF-α, IL-1β, and IL-6 induced by cisplatin and thus shows protective effects against cisplatin-induced ototoxicity (Altun et al., 2014). MR16-1 is an anti-IL-6 receptor neutralizing antibody that can suppress the effect of IL-6. After noise injury, blocking IL-6 signaling with MR16-1 significantly reduces the activation of cochlear macrophages in the spiral ganglion and reduces loss of SGNs, but does not lead to changes in the organ of Corti. Also, inhibiting IL-6 signaling improves low-frequency ABR thresholds in response to HLN exposure (Wakabayashi et al., 2010). Thus, MR16-1 has a protective effect in the noise-damaged cochlea.

Etanercept, a TNF-α inhibitor, significantly improves cochlear microcirculation, reduces the number of cells infiltrating into the cochlea and cochlear fibrosis, and protects against hearing loss after cochlear injury (Satoh et al., 2002; Wang et al., 2003; Arpornchayanon et al., 2013; Dhukhwa et al., 2019). Anti-ICAM-1antibodies (anti-ICAM-1Ab) may have preventive effects on cochlear damage as assessed by changes in auditory threshold and cochlear blood flow (Seidman et al., 2009). However, deficiency in IL-1β does not show any significant reduction in threshold shifts at any examined frequency, nor does it have any effect on macrophages entering the cochlea (Mizushima et al., 2017).

### Mesenchymal Stem Cells Regulate the Differentiation of Macrophages Into M1 and M2 Types

In recent years, stem cell application has emerged as a promising treatment modality for various auditory disorders. Preclinical animal studies have shown that mesenchymal stem cells (MSCs) can be used to treat SNHL (Lee et al., 2018; Chorath et al., 2019) because they have neuroprotective, anti-apoptotic, and anti-inflammatory properties. MSCs can interact with a variety of immune cells including T cells and macrophages, and they exert immunosuppressive properties (Zhou et al., 2011), which indicates that MSCs might be a novel therapeutic method for treating inner ear disorders such as HC damage caused by inflammation.

Human umbilical cord (UC) and BM MSCs have been used in the treatment of SNHL (Ma et al., 2016; Lee et al., 2018). MSCs can be found in the stria vascularis, the perilymphatic space, the organ of Corti, the basilar membrane, the spiral ganglion, and around the cochlear nerve fibers after transplantation (Jang et al., 2015; Ma et al., 2016), and Lee et al. (2018) verified that it is safe to transplant autologous BM MSCs into SNHL patients. The administration of MSCs does not lead to the generation of any oxidative stress, the production of proinflammatory cytokines, or the activation of apoptosis (Eshraghi et al., 2020), and thus the MSCs have no significant side effects on hearing thresholds (Mittal et al., 2020).

Recent studies have also demonstrated that MSC-based therapy has a remarkable effect on neurodegenerative diseases (Lee et al., 2009; Karussis et al., 2010; Glavaski-Joksimovic and Bohn, 2013; Hajivalili et al., 2016), which is in large part due to the immunomodulatory effects mediated by the interaction between MSCs and macrophages (Uccelli et al., 2008). It is challenging to distinguish between M1 and M2 macrophages (especially *in vivo*), because the two states are plastic. A series of studies have suggested that MSCs are able to regulate the differentiation of macrophages to the M2 type (Braza et al., 2016; Chiossone et al., 2016; Luz-Crawford et al., 2016), and considerable evidence suggests that the activation and maturation of innate immune cells can be inhibited by MSC-derived soluble factors, such as PGE_2_ and TSG-6, which can cause monocytes or M1 macrophages to convert into IL-10-expressing M2 populations (Chiossone et al., 2016; Ko et al., 2016). Preclinical studies have shown promising results in the application of MSCs for the treatment of pulmonary disorders, with MSCs promoting M2 macrophages (Gu et al., 2015). In a rat model of type 2 diabetes, intravenous administration of UC-MSCs reduced insulin resistance by promoting the M2 phenotype (Xie et al., 2016), and MSCs are also capable of converting microglia from a detrimental pro-inflammatory phenotype to a neuroprotective anti-inflammatory phenotype in the CNS by activating CX3CL1/CX3CR1 signaling (Giunti et al., 2012). Taken together, these results suggest that MSCs can suppress inflammatory macrophages, which makes the transplantation of MSCs very promising for treating SNHL.

## Concluding Remarks

Tissue-resident macrophages are widely distributed in different anatomical sites of the cochlea. When the cochlea suffers from injury, monocytes in the circulatory system migrate into the cochlea and transform into mature macrophages, which combine with tissue-resident macrophages to participate in immune responses. After acoustic and ototoxic injury, or in aged cochleae, cochlear HC loss is regarded as the initial damage. HC injury alone is sufficient to attract macrophages into the sensory epithelium and spiral ganglion (Kaur et al., 2015). The CX3CL1/CX3CR1 and CCL2/CCR2 signaling pathways have been shown to play a major role in recruiting macrophages into the cochlea. However, these two chemokines are still not enough to explain the detrimental and beneficial effect of the macrophage on auditory structure when CX3CR1 and CCR2 are knocked out. Current evidence suggests that macrophages also play a role in regenerating HCs in addition to damaging HCs after activation. At the same time, they also have the function of protecting SGNs and ribbon synapses after injury. The question, then, is why do macrophages have different effects on different structures of the cochlea? Studies have shown that tissue macrophages play an important role in early and acute injury, while recruited macrophages have an essential function in late and chronic injury. The differences in phenotype and activity between newly recruited macrophages and resident macrophages are still unclear. Future studies should explore the recruitment signaling pathways in more detail, determine the specific functions of cochlear macrophages under physiological conditions, and distinguish between resident macrophages and infiltrated macrophages. Even more importantly, it would be of great interest to clarify how macrophages perform their different functions when they enter different structures of the cochlea. Identifying the recruitment signaling pathways and cochlear macrophage functions will be a pivotal step to revealing new targets for the prevention and treatment of cochlear injury.

Damaged cochlear cells release DAMPs, which can activate PRR on the surface of macrophages. The PRR activation results in the production of inflammatory cytokines through the activation of a series of downstream signaling pathways. At present, the production of inflammation has been extensively studied, while the resolution of inflammation still needs to be further explored. Inhibiting inflammatory responses or promoting the transformation of macrophages to the M2 phenotype is necessary for tissue repair after stress. In future studies, deeper understanding of the biological process of macrophages and exploring the method of regulating the migration and function of macrophages will benefit patients suffering from SNHL.

## Author Contributions

All authors listed have made a substantial, direct and intellectual contribution to the work, and approved it for publication.

## Conflict of Interest

The authors declare that the research was conducted in the absence of any commercial or financial relationships that could be construed as a potential conflict of interest.

## Publisher’s Note

All claims expressed in this article are solely those of the authors and do not necessarily represent those of their affiliated organizations, or those of the publisher, the editors and the reviewers. Any product that may be evaluated in this article, or claim that may be made by its manufacturer, is not guaranteed or endorsed by the publisher.
